# Diagnostic accuracy of interleukin-6 (IL-6) as a significant biomarker in late-onset neonatal sepsis: an updated systematic review and meta-analysis

**DOI:** 10.1007/s00431-025-06409-w

**Published:** 2025-09-02

**Authors:** Amani N. Alansari, Mohamed Sayed Zaazouee, Alaa Ahmed Elshanbary, Salma Mani, Marwa Messaoud

**Affiliations:** 1 Department of Pediatric Surgery, Hamad Medical Corporation, Doha, Qatar; 2 Faculty of Medicine, Al-Azhar University, Assiut, Egypt; 3 Faculty of Medicine, Alexandria University, Alexandria, Egypt; 4 Pediatric Surgery Department, Fattouma Bourguiba University Hospital, Monastir, Tunisia; 5 Faculty of Medicine of Monastir, University of Monastir, Monastir, Tunisia; 6 Research Laboratory of Congenital Anomalies and Childhood Cancer LR12SP13, University of Monastir, Monastir, Tunisia

**Keywords:** Biomarker, Diagnostic accuracy, Interleukin-6, IL-6, Late-onset neonatal sepsis

## Abstract

Late-onset neonatal sepsis (LONS) remains a leading cause of morbidity and mortality in neonates, particularly among preterm and low birth weight infants. Interleukin-6 (IL-6) has emerged as a promising biomarker for early detection of LONS, yet the diagnostic performance of IL-6 remains inconsistent across studies. This meta-analysis aimed to evaluate the overall diagnostic accuracy of IL-6 in detecting LONS and to assess the certainty of evidence using the GRADE approach. A comprehensive search of five databases was conducted up to July 15, 2025. Twenty-two studies were eligible for inclusion, of which 20 (*n* = 3527 neonates) were included in the meta-analysis. A bivariate random-effects model was used to estimate pooled sensitivity and specificity, and the area under the SROC curve was used to assess overall diagnostic accuracy. Pooled sensitivity and specificity were 85.2% (95% CI: 80.0–89.3%) and 84.1% (95% CI: 77.5–89.0%), respectively. The positive and negative likelihood ratios were 5.43 and 0.18, with a diagnostic odds ratio of 31.4. The AUC was 0.91, indicating excellent diagnostic accuracy. Subgroup and sensitivity analyses confirmed the robustness of the findings, and no significant threshold effect was detected. However, suspected publication bias was noted. The GRADE assessment rated the certainty of evidence as moderate. *Conclusion*: IL-6 demonstrates high diagnostic accuracy for LONS and may serve as a valuable tool for early identification, though standardization of cutoff thresholds and further validation studies are warranted.

**Table:** 

**What is Known:** • *IL-6 is an early-rising biomarker for late-onset neonatal sepsis (LONS), but its diagnostic accuracy varies across studies.*
**What is New:** • *This meta-analysis used a bivariate model to estimate IL-6 performance, yielding high pooled sensitivity (85.2%) and specificity (84.1%).*

**Supplementary Information:**

The online version contains supplementary material available at 10.1007/s00431-025-06409-w.

## Introduction

Neonatal sepsis is one of the leading contributors to morbidity and mortality in newborns, being responsible for 13% of all neonatal mortality, and 42% of deaths in the first week of life [1, 2]. Despite the advances in neonatal care, neonatal sepsis is widely encountered in neonatal intensive care units (NICU). Its incidence ranges from 1 to 10 per 1000 live births worldwide, with the actual caseload being significantly higher in low-resource settings [3]. Early diagnosis is crucial to avoid the devastating consequences, including multi-organ dysfunction, long-term neurodevelopmental impairment, and increased mortality [4]. Neonatal sepsis is classified as early-onset (EONS) or late-onset (LONS), based on whether it occurs before or after 72 h of life [5]. Unlike early-onset cases, which usually result from vertically transmitted group B *Streptococcus* (GBS) or *Escherichia coli* (*E. coli*), which are predictable and covered by standard empirical antibiotic therapy, LONS is usually acquired postnatally from the hospital environment or the infant’s own acquired microbiota [1, 6]. Blood culture remains the gold standard for diagnosis; yet, its sensitivity is influenced by factors such as the volume of blood collected, prior antibiotic exposure, the severity of bacteremia, and the laboratory’s technical capabilities [7]. Therefore, in the context of neonatal sepsis, there is a critical need for early-rising and accurate biomarkers to enable prompt and reliable diagnosis, particularly in cases where culture results are delayed or inconclusive. These biomarkers would help in risk stratification, guide in the early intervention, and ultimately improve neonatal outcomes [8].

Many biomarkers have been explored for the diagnosis of neonatal sepsis. C-reactive protein (CRP) and procalcitonin (PCT) are among the widely used markers; both, however, are not without limitations [7]. CRP begins to surge around 10–12 h after infection onset, peaking at 24–48 h, making it suboptimal for early diagnosis [9, 10]. PCT is influenced by conditions like stress implying birth and by perinatal asphyxia due to its association with non-infectious inflammatory states [11, 12]. Other biomarkers, including interleukins, hepcidin, and CD64, were investigated. They showed promise, but their diagnostic performance varies in different studies [13–15]. Interleukin-6 (IL-6) is emerging as a candidate for a possible early marker of neonatal sepsis. It plays a key role in innate immunity, stimulating acute-phase reactions against infection and modulating pro-inflammatory cascades [16]. Its most marked distinguishing feature is the quick rise after the onset of infection [17]. Nevertheless, the diagnostic performance of IL-6 remains quite controversial across various studies, from excellent sensitivity and specificity to inadequate sensitivity and high false-positive rates [18–21]. Meeting these gaps, we conducted this meta-analysis to pool the available evidence about the diagnostic performance of IL-6 in neonatal sepsis, particularly in LONS.

### Methods

This meta-analysis was conducted in accordance with the Preferred Reporting Items for Systematic Reviews and Meta-Analyses (PRISMA) guidelines [22].

### Search strategy

A comprehensive search was conducted in PubMed, Embase, Scopus, the Cochrane Library, and Web of Science up to July 10, 2025. Additional manual screening was performed through Google Scholar, ClinicalTrials.gov, and relevant grey literature sources. All retrieved records were imported into EndNote, and duplicates were removed. Two independent reviewers screened titles and abstracts for relevance based on predefined inclusion and exclusion criteria. Full-text articles of potentially eligible studies were then assessed for final inclusion. Any discrepancies between reviewers were resolved through discussion, with a third reviewer consulted if necessary. Full details of the database-specific search strategies and number of retrieved records are provided in Table S1.

#### Eligibility criteria

Studies assessing the diagnostic performance of IL-6 in late-onset neonatal sepsis, defined as sepsis occurring after 72 h of life, were considered for inclusion. Eligible studies had to report sensitivity and specificity or provide raw data on true positives, false positives, true negatives, and false negatives. Studies were excluded if they were non-English, case series or case reports, not limited to neonates, focused on sepsis within the first 72 h of life, or lacked diagnostic accuracy data.

### Data extraction and quality assessment

Three independent reviewers extracted relevant data, which were then summarized and tabulated. The extracted information included study details such as author, year of publication, study design, country, neonatal age, time frame of the LONS, number of patients recruited, clinical presentation, and main findings. Data on diagnostic testing included the reference standard, test used for IL-6 measurement, IL-6 cutoff value, sensitivity, specificity, area under the curve (AUC), positive predictive value (PPV), and negative predictive value (NPV). In studies combining IL-6 with other biomarkers, we extracted the data corresponding to IL-6 alone. The methodological quality of the included studies was assessed using the Quality Assessment of Diagnostic Accuracy Studies-2 (QUADAS-2) tool, which evaluates four domains: patient selection, index test(s), reference standard, and flow and timing [23]. Additionally, the certainty of the evidence for pooled estimates of sensitivity and specificity was evaluated using the GRADE (Grading of Recommendations Assessment, Development and Evaluation) approach for diagnostic test accuracy [24]. Both assessments were performed independently by two reviewers, with disagreements resolved through consensus or by consulting a third reviewer.

### Statistical analysis

For the included studies, per-study estimates of sensitivity and specificity of IL-6 were provided in a paired forest plot. Our analysis stems from the true/false positive/negative frequencies reported in included studies (no missing values found). Due to the special characteristics of diagnostic test accuracy meta-analysis, pooled estimates (and 95% confidence intervals) of test performance metrics were obtained from a bivariate model [25]. Bivariate modelling takes into account the “threshold effect”—the negative correlation between sensitivity and specificity—which makes it superior to separately pooling sensitivity and specificity through random-effects models [26]. Summary ROC (SROC) curves were also fit. Being an ongoing field of research and an area of daily technological advancements, methods to perform sensitivity analysis and assess for publication bias given the output of the bivariate model are still being developed. The trim and fill method is a robust approach to investigate publication bias [27]. It is based on the funnel plot and is favorable over other available methods in diagnostic test accuracy meta-analysis [28]. To address the sensitivity of our findings to potential publication bias, we utilized left-sided trim and fill to identify influential cases in terms of diagnostic test accuracy. Additionally, we conducted sensitivity analyses based on study-level risk of bias (QUADAS-2 domains) and study design (e.g., prospective vs. retrospective) to evaluate the robustness of the pooled estimates under varying methodological quality. Analysis and reporting were done using the R programming language for statistical computing, mainly the mada package [29, 30]. Trim and fill functionality was imported from the metafor package [31].

## Results

### Search results

**Fig. 1:**
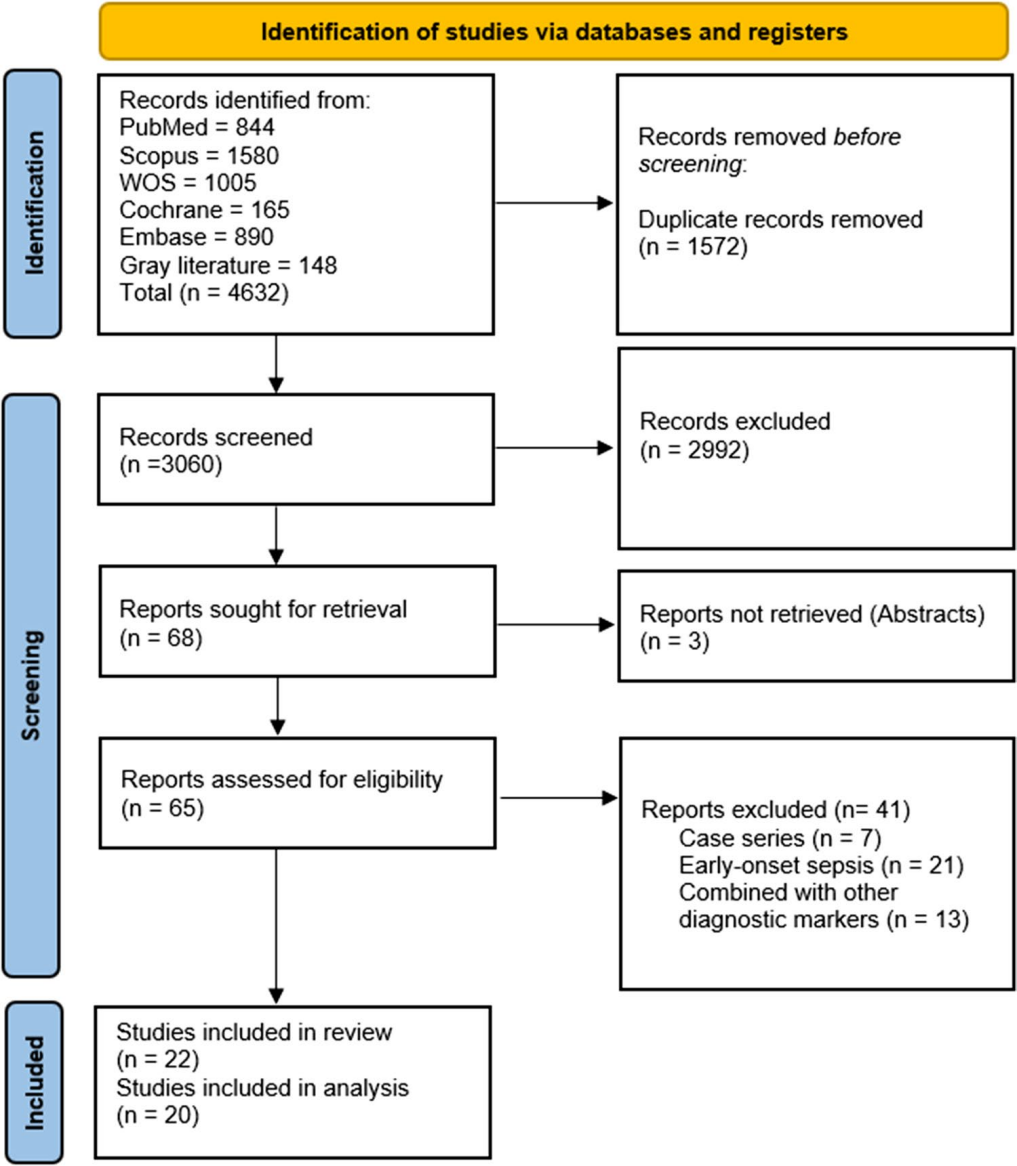
The PRISMA flowchart

A total of 4632 records were identified through the database search. After removing duplicates, 3060 records remained for title and abstract screening. Of these, 65 full-text articles were assessed for eligibility based on the predefined inclusion criteria. Ultimately, 22 studies were included in this systematic review, with 20 studies eligible for meta-analysis. The study selection process is illustrated in the PRISMA 2020 flow diagram (Fig. 1).

### General characteristics

A total of 22 studies were included [18–21, 32–50]. The majority were prospective cohort studies, with some case–control studies and retrospective analyses. The studies were conducted in diverse regions, including China, India, Turkey, Bulgaria, Austria, France, Greece, USA, Brazil, Egypt, and Indonesia. Their general characteristics are summarized in Table 1.

**Table 1:** Summary of the included studies

ID	Study design	Country	Neonate’s age	LOS time frame	Patients recruited	Clinical presentation	Main results
Chen 2025	Case–control study	China	13.92 ± 5.04 days (term) and 14.39 ± 3.85 days (premature) infants​	> 72 h	A total of 240 neonates were recruited (120 with neonatal sepsis—60 premature and 60 term infants—and 120 non-infected controls—60 premature and 60 term infants)​	Abnormal body temperature, respiratory changes, lethargy, feeding difficulties, and hemodynamic instability	IL-6 was found to be a reliable diagnostic biomarker for neonatal sepsis The combination of IL-6 with other biomarkers (e.g., CRP, PCT) improved diagnostic performance​
Santos 2024	Multicenter prospective cohort study	Brazil	From 3 days onward	> 72 h	53 neonates with blood culture-proven LOS 22 healthy full-term controls sampled on day 3 before hospital discharge​	Fever, cardiovascular, respiratory, gastrointestinal, and neurological issues, with the most common pathogens being coagulase-negative Staphylococcus and Staphylococcus aureus	IL-6 demonstrated excellent diagnostic and prognostic accuracy for (LOS), distinguishing survivors from non-survivors with an AUC > 0.90 at all-time points, whereas ADM and CRP showed limited prognostic value​
Goyal 2024	Prospective cohort study	India	N.D	> 72 h	82 neonates with clinically suspected sepsis	Respiratory distress (71.95%), feeding intolerance (35.37%), and hemodynamic instability (30.49%)	CRP had the highest diagnostic accuracy (AUC 0.73), followed by PCT (AUC 0.65) and IL-6 (AUC 0.55). IL-6 had high sensitivity but low specificity
Rupin 2024	Prospective cohort study	USA	Within 7 days of birth	> 72 h	118 VLBW infants (54 analyzed) Mean gestational age = 25.9 ± 1.9 weeks Mean birth weight = 803 ± 222 g	Tachycardia, feeding intolerance, pnea, respiratory distress, and abdominal distension​	IL-6 had 100% sensitivity and 78% specificity for diagnosing Gram-negative sepsis or NEC at a threshold of 200 pg/mL, and combining it with the Pulse Oximetry Warning Score improved predictive accuracy​
Gatseva 2023	Prospective cohort study	Bulgaria	Neonates with a stay in the NICU exceeding 72 h	> 72 h	Total: 60 newborns Group 1: Symptomatic and infected (n = 21) Group 2: Symptomatic but uninfected (n = 20) Group 0: Asymptomatic controls (n = 19)	Temperature instability, apnea, tachypnea, tachycardia, dyspnea, hyper-/hypothermia, feeding difficulties, and irritability​	IL-6 and (PCT) were the most sensitive markers for early LOS diagnosis; combining IL-6, PCT and platelet count (PLT) increased diagnostic accuracy (AUC = 0.880)
Kung 2023	Retrospective cohort study	Austria	> 7 days	> 7 days	1,695 neonates in total, including 752 very-preterm infants and 701 very-low-birthweight infants	Fever or hypothermia, respiratory distress, poor feeding, lethargy, pale or mottled skin, and jaundice, along with abnormal blood work such as elevated white blood cell count and CRP	IL-6 cut-off values were defined for diagnosing culture-confirmed sepsis with good sensitivity and specificity
Pons 2023	prospective, multicenter cohort study	France	7–178 days	> 72 h	234 hospitalized neonates with suspected LOS were enrolled; 230 had analyzable samples. Of these, 51 were classified as infected, 153 as not infected, and 26 were unclassified​	Fever, tachycardia, capillary refill time > 3 s, gray/pale skin complexion, apnea/bradycardia, and respiratory distress	IL-6 was one of the best-performing biomarkers with an AUC of 0.864, and using biomarkers could have avoided up to 64% of unjustified antibiotic use​
Cui 2021	case–control study	China	14.32 ± 9.74	> 72 h	The study enrolled 126 preterm infants, with 89 infants in the sepsis group (47 confirmed sepsis and 42 clinical sepsis) and 31 infants in the control group	Respiratory distress, Tachycardia or bradycardia, Poor perfusion, Feeding intolerance, Jaundice and abnormal body temperature	The study found that IL-6, CRP, and PCT are reliable indicators for early diagnosis of nosocomial infections in preterm infants, with IL-6 peaking 6 h after infection onset and returning to normal 24–48 h after infection control and combining PCT with IL-6 or CRP improving diagnostic accuracy
Değirmencioğlu 2019	Prospective, case–control study	Turkey	4 to 60 days	> 72 h	26 preterm infants with culture-proven LOS 29 preterm infants without sepsis (control group)	Respiratory (tachypnea, apnea, increased ventilatory support) Metabolic (feeding problems, temperature instability, metabolic acidosis) Circulatory (hypotension, poor perfusion, bradycardia) Neurologic (lethargy, irritability)	Presepsin showed high diagnostic accuracy for culture-proven late-onset sepsis in preterm infants with 88.9% sensitivity and 88.9% specificity at a cutoff value of 823 ng/ml, while fetuin-A levels were not significantly different between septic and control groups
Saldir 2015	Prospective cohort study	Turkey	99 ± 24 h	> 72 h	Total = 50 neonates 30 septic neonates (6 with proven sepsis, 24 with probable sepsis) 20 nonseptic neonates (control group)	Feeding intolerance, respiratory distress, hypotension, and clinical deterioration​	Elevated levels of sTREM-1 and endocan, along with IL-6 ratio, are effective for diagnosing neonatal sepsis, with sTREM-1 showing the highest diagnostic accuracy
Tunc 2014	Prospective cohort study	Turkey	98.9 ± 22.2 h	> 72 h	30 septic neonates (5 with proven sepsis, 25 with probable sepsis) 20 non-septic neonates (admitted for conditions other than sepsis)	Temperature instability, apnea, tachycardia/bradycardia, hypotension, feeding intolerance, abdominal distension, and clinical deterioration	Elevated serum levels of CXCR4 and CXCL12 are significantly associated with neonatal sepsis and decrease after treatment, making them potential biomarkers for early diagnosis
Lusyati 2013	Prospective cohort study	Indonesia	Older than 72 h	> 72 h	Proven Sepsis (PS) group – 18 infants Clinical Sepsis group – 25 infants Control group – 34 infants	Temperature instability, apnea, bradycardia, desaturations, respiratory dysfunction, hypotension, poor capillary refill, feeding intolerance, abdominal signs of NEC, hyperglycemia, metabolic acidosis, seizures	IL-6, IL-8, IL-15 and TNF-α are potentially good markers to differentiate proven LOS from clinical sepsis, with IL-6 and IL-15 showing the best sensitivity and specificity at 12 and 24 h
Maaboud 2012	Case–control study	Egypt	3–30 days	> 72 h	48 full-term neonates with late-onset sepsis and 40 healthy neonates as controls	Feeding intolerance, lethargy, irritability, temperature instability, prolonged capillary refill, jaundice, tachypnea, recurrent apnea, tachycardia, or bradycardia	IL-6 and calprotectin levels were significantly elevated in neonates with late-onset sepsis, with IL-6 showing high sensitivity (89%) and specificity (92.2%) at a cutoff of 160 pg/mL
Raynor 2012	Prospective cohort study	USA	> 3 days old	> 72 h	A total of 226 plasma samples were obtained from 163 NICU patients with suspected sepsis. The gestational age of the infants was 28.7 ± 4.7 weeks, and the birth weight was 1,311 ± 861 g (mean ± SD)	Gastrointestinal (feeding intolerance, necrotizing enterocolitis), respiratory (apnea, distress), CNS (temperature instability, lethargy), skin infections, and abnormal lab findings (hyperglycemia, acidosis, bandemia)​	Cytokine screening using IL-6, IL-8, TNF-α had 100% sensitivity and 69% Positive predictive value for detecting Gram-negative bacteremia in NICU patient
Hotoura 2011	Prospective cohort study	Greece	> 3 days old	> 72 h	82 preterm neonates classified into: Sepsis group: 17 neonates with confirmed sepsis (positive blood culture with symptoms) Suspected infection group: 25 neonates with infection symptoms but negative cultures Control group: 40 infection-free neonates	Poor peripheral infusion, capillary refill > 3 s, abnormal skin color, hypotension, temperature instability, hypoglycemia, feeding issues, respiratory distress, and seizures	IL-6 > 30 pg/ml combined with CRP > 10 mg/l was the most accurate predictor of sepsis, with sensitivity 1.0 and specificity 0.96
Sarafidis 2010	Prospective cohort study	Greece	> 3 days of life	> 72 h	Total: 52 neonates 31 infected neonates (22 with confirmed sepsis + 9 with possible sepsis) 21 non-infected neonates	Temperature instability, respiratory distress, gastrointestinal dysfunction, cardiovascular changes, and abnormal lab results (metabolic acidosis, thrombocytopenia, leukopenia)​	Serum sTREM-1 and IL-6 levels were significantly elevated in infected neonates, but sTREM-1 alone was not superior to IL-6 for diagnosing late-onset sepsis
Ng 2007	prospective cohort study	China	≥ 72 h old	> 72 h	Total suspected infection episodes: 155 Infected group (Group 1): 44 episodes (36 infants) Noninfected group (Group 2): 111 episodes (50 infants)	Temperature instability, apnea, respiratory distress, GI symptoms (abdominal distention, bloody stools), cardiovascular dysfunction, abnormal lab findings (metabolic acidosis, thrombocytopenia, leukopenia/leukocytosis)	IL-6 (cutoff 26.1 pg/mL) showed 82% sensitivity, 82% specificity, AUC = 0.88, making it a useful marker for diagnosing late-onset neonatal sepsis
Maciolek 2006	Prospective cohort study	Netherlands	0 to 60 days	> 72 h	111 infants in total (92 NICU patients and 19 pediatric ward patients)	Fever, temperature instability, irritability, lethargy, apnea, abdominal distension, respiratory distress, poor feeding, diarrhea, etc	IL-6, IL-8, and PCT outperformed CRP in diagnosing neonatal sepsis and differentiating bacterial from viral infections
Arnon 2005	Prospective cohort study	Israel	5–36 days	> 72 h	Total: 116 infants Proven sepsis group = 23 Clinical sepsis group = 15 non-sepsis group = 78 (37 preterm infants without sepsis and 41 healthy controls)	Pallor, poor skin perfusion, apnea, bradycardia, hypotension, respiratory dysfunction, and metabolic abnormalities (acidosis, hyperglycemia)​	Serum amyloid A was a reliable early marker for late-onset sepsis in preterm infants, with high sensitivity (95–100%) (97–100%) in the first 24 h​
Gonzalez 2003	Two-phase study: retrospective (S1) and prospective (S2) cohort	USA	≥ 72 h old	> 72 h	S1: Retrospective review of 48 episodes of culture-proven late-onset sepsis from 1991–1998 S2: 27 infants enrolled in a prospective study, with 8 confirmed sepsis cases and 19 non-sepsis cases	Apnea, bradycardia, respiratory distress, increased ventilatory requirements, desaturations, and gastric residuals	IL-6 levels on days 0 and 1 were significantly higher in infants with confirmed sepsis, making it a potential early marker for diagnosing late-onset neonatal sepsis
Ng 2002	prospective cohort study	China	At least 72 h old	> 72 h	80 VLBW infants with 127 episodes of suspected clinical sepsis. Of these, 37 episodes were confirmed as infections	Unstable temperature, hypotension, recurrent apnea, and severe desaturation	Neutrophil CD64 expression was found to be a highly sensitive marker for diagnosing late-onset nosocomial infection in VLBW infants, with sensitivity and negative predictive values exceeding 95% at 0 and 24 h after sepsis evaluation
Ng 1997	Prospective cohort study	China	> 72 h of age	> 72 h	Total of 101 episodes of suspected clinical sepsis were investigated in 68 (VLBW) infants 45 episodes were confirmed as infections	Unstable temperature, lethargy, feeding intolerance, respiratory issues, cardiovascular instability (bradycardia, tachycardia), and hematologic changes (thrombocytopenia, leukopenia)​	IL-6 had the highest sensitivity (89%) and NPV(91%) for detecting late-onset sepsis on day 0, while CRP was most effective after 24–48 h

*CRP* C-reactive protein, *PCT* procalcitonin, *IL-6* interleukin-6, *VLBW* very low birth weight, *LOS* late onset-sepsis, *AUC* area under curve, *ADM* adrenomedullin, *NICU* neonatal intensive care unit, *NEC* necrotizing enterocolitis, *CXCR4* chemokine receptor type 4, *CXCL12* chemokine receptor ligand 12, *TNF-α* tumor necrosis factor-alpha, *sTREM-1* soluble triggering receptor expressed on myeloid cells-1, *NS-PI* neonatal sepsis-premature infant, *NS-TI* neonatal sepsis-term infant, *N.D* not determined

Table 2 summarizes the details of diagnostic testing and the accuracy parameters. All studies used blood culture-confirmed sepsis as the reference standard. Cutoff values for IL-6 varied widely, with the studies by Rupin et al. and Maaboud et al. using the highest values [19, 41]. At a cutoff of 7 pg/mL, Tunc et al. reported high sensitivity (93.3%) and specificity (95%) [49]. Similarly, at 10 pg/mL, Santos et al. reported a sensitivity of 96.2% and a specificity of 90.9% [36]. For 18–31 pg/mL, sensitivity ranged from 75 to 89%, while specificity varied between 68 and 96% [38, 42]. Higher cutoffs, such as 65.98–99.6 pg/mL, showed sensitivity between 68% and 87.6%, with specificity up to 91.3% [34, 50]. For very high cutoffs (≥ 130 pg/mL), Raynor et al. reported 100% sensitivity but poor specificity (28%), while Rupin et al. at 200 pg/mL maintained 100% sensitivity with improved specificity (78%) [19, 46]. On the contrary, Maaboud et al. used a cutoff value of 160 pg/mL, reporting a lower sensitivity (89%) and an excellent specificity (92.2%) [41]. Notably, one study reported 100% sensitivity with the lowest cutoff value (7 ng/mL), but with low specificity (34.48%) [18].

**Table 2:** Details of diagnostic testing

ID	Standard reference	Test used	IL-6 cutoff	Sensitivity (%)	Specificity (%)	AUC (95% CI)	PPV (%)	NPV (%)
Chen 2025	Positive blood culture​	ELISA	44.487 pg/mL for NS-PIs and 43.422 pg/mL for NS-TIs	68% for NS-PIs,60% for NS-TIs	95% for NS-PIs, 96% for NS-TIs	0.817 (NS-PIs) and 0.801 (NS-TIs)​	0.932 for NS-PIs, 0.967 for NS-Tis	0.750 for NS-PIs, 0.784 for NS-Tis
Santos 2024	Positive blood cultures	ELISA	10 pg/mL	96.20%	90.9%	0.985	93.50%	45.50%
Goyal 2024	Positive blood culture	point-of-care (POC) testing with the AFIAS-6	7 pg/mL	100%	34.48%	0.55	36.67%	100%
Rupin 2024	Positive blood culture	Customized multiplex Luminex® assay	200 pg/mL	100%	78%	0.68	75%	100%
Gatseva 2023	Positive culture	ELISA	≥ 27.5 pg/mL	78%	70%	0.752	58.33%	85.53%
Kung 2023	Blood culture	Vitros 250, Ortho Clinical Diagnostics, and Cobas 6000, Roche)	30 pg/mL	75%	82%	0.9	60%	90%
Pons 2023	Positive blood culture	ELLA Automated Immunoassay System	N.D	0.89	100%	0.864 (0.798–0.929)		
Cui 2021	Positive blood culture	ELISA	99.6 pg/mL	87.60%	91.30%	0.888	96.30%	71.80%
Değirmencioğlu 2019	Positive blood culture	IMMULITE 1000 analyzer	23.22 pg/mL	94.40%	78.20%	0.959	75%	95.40%
Saldir 2015	Positive blood or CSF cultures for microorganisms	ELISA	7 ng/mL	93.30%	95%	0.96 (95% CI = 0.908–0.998, p < 0.001)	96.60%	90.50%
Tunc 2014	Positive blood or CSF cultures for microorganisms	ELISA	7 pg/mL	96.70%	95%	0.97 (95% CI = 0.918–0.998)	96.70%	95%
Lusyati 2013	Blood culture (BACTEC method)	Invitrogen-immunoassays-Luminex™ 100	> 93	72.22%	72.22%	N.D	N.D	N.D
Maaboud 2012	Blood culture	ELISA	160 pg/mL	89%	92.20%	N.D	96.70%	84.80%
Raynor 2012	Positive blood culture	multiplex antibody-coated bead array with dual-laser fluorometric detection	< 130 pg/mL	100%	93%	N.D	38%	100%
Hotoura 2011	Positive blood culture	ELISA	30 pg/mL	100%	74%	0.95	40%	100%
Sarafidis 2010	Positive blood culture for microbes or fungi	ELISA	65.98 pg/mL	80%	81%	0.892 (95% CI: 0.808–0.976)	86%	74%
Ng 2007	Positive blood cultures	Cytometric Bead Array Kit and Flow Cytometry	26.1 pg/mL	82%	82%	0.88	64%	92%
Maciolek 2006	Positive blood culture for bacterial infection or RT-PCR for enterovirus	Fully automated chemiluminescence assay	≥ 60 pg/mL	68%	76%	N.D	78%	65%
Arnon 2005	Positive blood cultures	ELISA	31 pg/mL	78%	89%	0.65 (0.35–0.76)	64%	88%
Gonzalez 2003	Blood cultures	ELISA	18 pg/mL	75%	68%	N.D	50%	87%
Ng 2002	Positive cultures (blood, CSF, urine)	ELISA	31 pg/mL	78%	92%	N.D	81%	91%
Ng 1997	Positive blood culture	ELISA	31 pg/mL	89%	96%	N.D	95%	91%

*ELISA* enzyme-linked immunosorbent assay, *IMMULITE1000* IMMULITE 1000’s enzyme-amplified chemiluminescence, *CSF* cerebrospinal fluid, *CRP* C-reactive protein, *PCT* procalcitonin, *IL-6* interleukin-6, *LOS* late onset-sepsis, *AUC* area under curve, *ADM* adrenomedullin, *PCR* polymerase chain reaction, *NS-PI* neonatal sepsis-premature infant, *NS-TI* neonatal sepsis-term infant, *N.D* not determined, *CI* confidence interval

Two studies were excluded from the meta-analysis due to their exclusive focus on Gram-negative sepsis, limiting their generalizability to the broader neonatal sepsis population. Rupin et al. (2024) evaluated 118 very low birth weight infants within the first week of life and reported that IL-6, at a cutoff of 200 pg/mL, achieved 100% sensitivity and 78% specificity for diagnosing gram-negative sepsis or necrotizing enterocolitis. The study emphasized the enhanced diagnostic accuracy when IL-6 was combined with the Pulse Oximetry Warning Score [19]. Raynor et al. (2012) assessed 226 samples from 163 NICU infants and demonstrated that cytokine screening (including IL-6) yielded 100% sensitivity and 69% PPV for Gram-negative bacteremia [46]. Both studies, while supporting the diagnostic utility of IL-6, were excluded due to their selective pathogen focus, which introduces threshold and spectrum biases not compatible with pooled estimates intended to reflect sepsis of all etiologies.

### Quality and certainty of evidence

The methodological quality of the included studies was assessed using the QUADAS-2 tool. Overall, 13 studies (59.1%) were judged to have low risk of bias, 7 studies (31.8%) had moderate risk, and 2 studies (9.1%) had high risk of bias. The most common concerns were related to the reference standard domain. Full domain-level assessments are presented in Table S2.

We also assessed the certainty of the evidence using the GRADE approach for diagnostic test accuracy. Certainty was rated as moderate for both sensitivity (true positives) and specificity (true negatives), mainly due to suspected publication bias detected through asymmetry in the trim-and-fill funnel plot. Other domains (risk of bias, indirectness, inconsistency, and imprecision) were not considered serious. Detailed GRADE ratings are provided in Table S3.

### Meta-analysis

#### Descriptive analysis

**Fig. 2:**
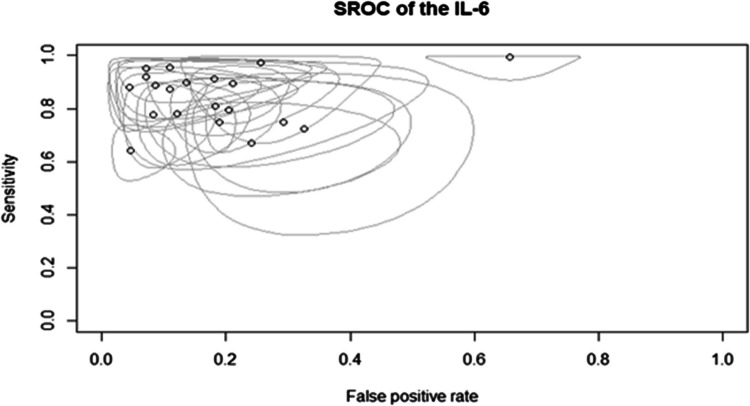
Summary ROC of diagnostic odds ratio as per included studies (n: 20 studies)

**Fig. 3:**
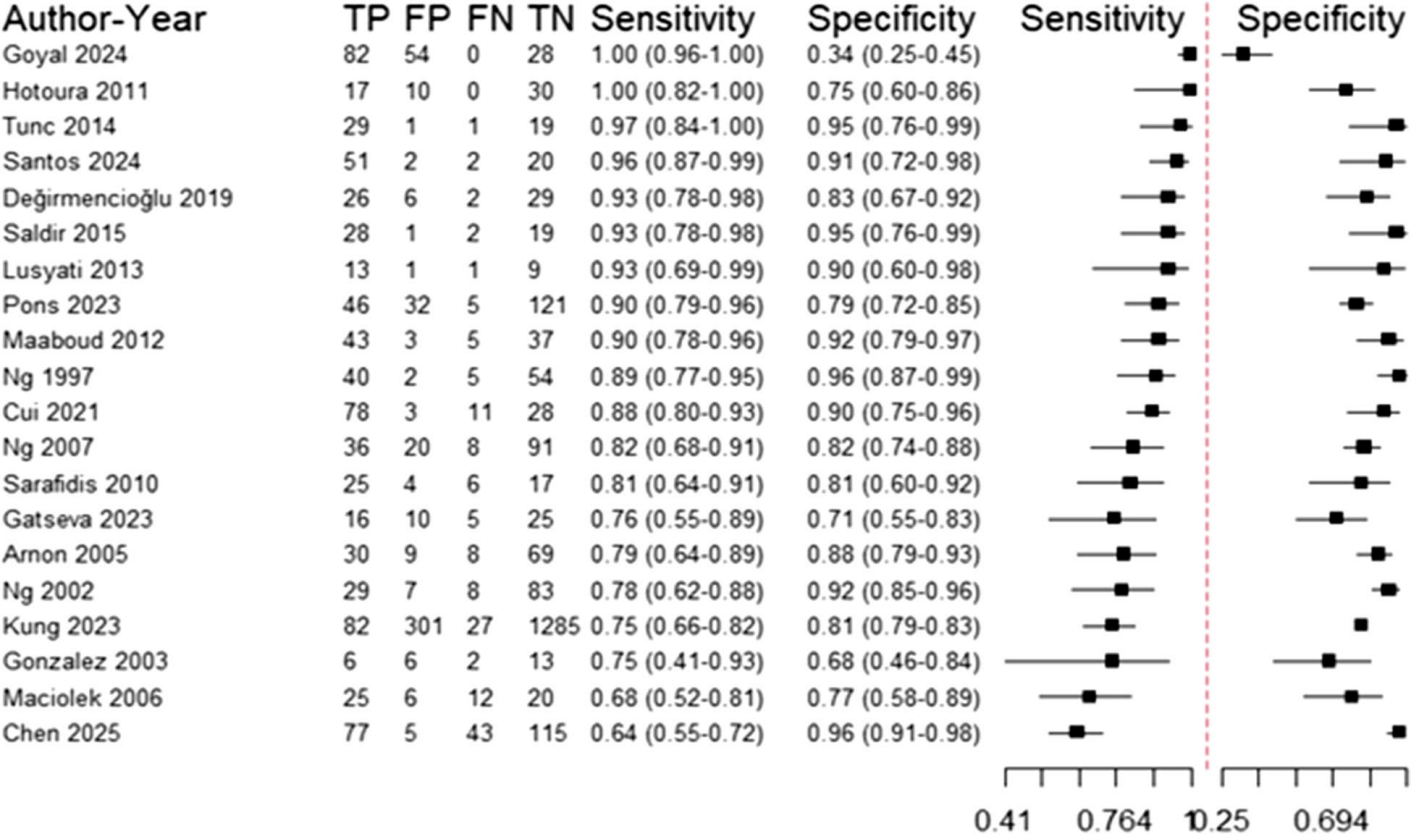
Forest plot included studies (n: 20 studies)

The current meta-analysis included a total of 20 studies with a total of 3527 recruited study participants. Table S4 shows the sensitivity, specificity, and diagnostic odds ratio reported per each study. Most studies reported very good to excellent sensitivity (> = 80%; 13/20 studies) as well as specificity (14/20 studies) with diagnostic odds ratio (DOR) being larger than 30 in most of the studies (15/20 studies). Such a remarkable diagnostic performance of IL-6 also yielded a notable pattern on the summary ROC (SROC) curve of included studies (Fig. 2) with a tendency towards the upper left part of the figure (high sensitivity and specificity region). The actual contingency table frequencies are presented in Fig. 3, with studies being arranged in a descending order of the reported sensitivity values in order to simplify the overall interpretation of our included data. Upon formal evaluation, no evidence of potential publication bias was detected (Spearman’s rho between sensitivity and false-positive rate: 0.074; *p*-value: 0.757).

#### Bivariate analysis

**Fig. 4:**
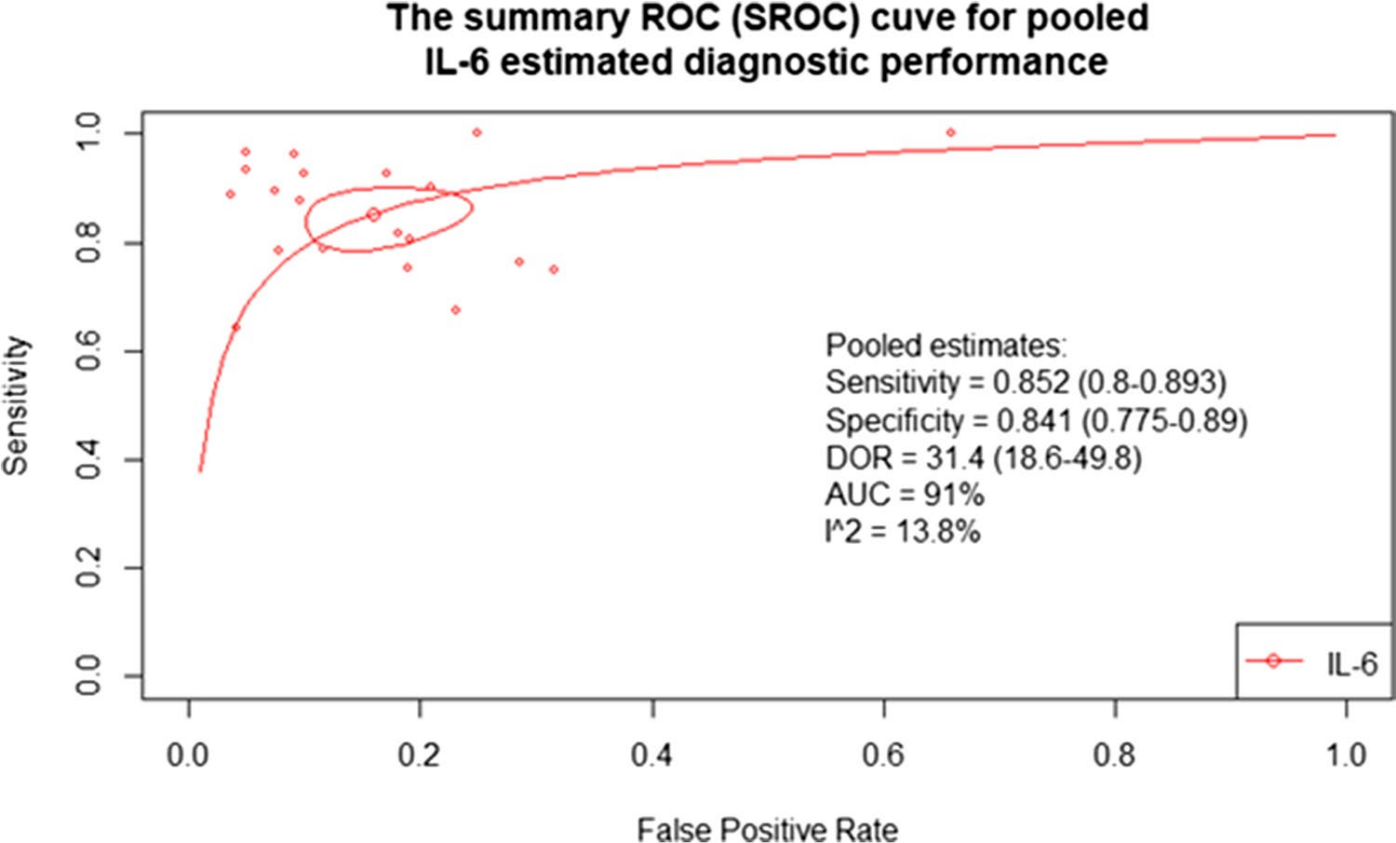
Summary ROC of pooled IL-6 diagnostic odds ratio

Table S5 presents findings of the bivariate model fit to pool the overall diagnostic test performance metrics of IL-6 after correcting for the potential threshold effect and correlating nature of the diagnostic performance metrics. The model pooled an estimated very good sensitivity of 85.2% (95% CI: 80% to 89.3%) with a similarly encouraging specificity of 84.1% (95% CI: 77.5% to 89%). Such an excellent diagnostic performance is also reflected upon the positive and negative likelihood ratios (LR + and LR-, respectively). Our analysis revealed a LR + of 5.43 (95% CI: 3.81 to 7.63) and a LR- of 0.18 (95% CI: 0.13 to 0.24), highlighting the diagnostic value of IL-6 in correct stratification of suspected cases. All these metrics may be summarized via the pooled DOR of 31.4 (95% CI: 18.6 to 49.8) and the 91% area under the curve coverage. Estimated heterogeneity was accepted (I^2: 13.8%). Figure 4 provides a visual stand-alone summary of these findings.

Table S5 also shows that although our included studies (n: 20) had a study with high ROB (n: 1) and 5 studies which are not prospective cohort (2 prospective case–control, 1 retrospective cohort, 1 retrospective case–control, 1 mixed retro- and prospective cohort), our pooled results were not sensitive to their exclusion. This is made clear due to the overlapping 95% CIs of all of the pooled sensitivity, specificity, LR +, LR-, and DOR. Moreover, the range of AUC and I^2 variation across the three models was minimal.

Additionally, the Fagan nomogram provided in Figure S1 goes a step further down the road. Given our pre-test probability of being septic among the cases recruited in our included studies and the excellent LR + and LR- performance, the post-test probability of having the disease becomes clearer. On the nomogram, the post-test probability that a case with high IL-6 levels suggestive of LONS increases to up to 67%. In contrast, the probability of being a LONS case after a negative IL-6 test becomes as small as 6%.

However, Figure S2 shows that potential publication bias is speculated. The trim and fill funnel plot estimated that several studies with comparatively small effect sizes could have been left unpublished/trimmed from the plot. The asymmetrical shape of the plot suggests that publication bias can’t be ignored.

## Discussion

The key step for managing neonatal sepsis is the early diagnosis and timely prompt initiation of treatment. Therefore, identifying a quick, sensitive, and specific biomarker is crucial for optimizing care. IL-6 rises sharply within 2 to 3 h in response to endotoxin and declines rapidly within 8 h, making it a valuable early marker for sepsis detection [51]. This meta-analysis pooled the diagnostic accuracy metrics of IL-6 in diagnosing LONS. Using a bivariate random-effects model, we estimated pooled sensitivity and specificity at 85.2% and 84.1%, respectively. To contextualize these results clinically, we utilized a Fagan nomogram to translate likelihood ratios into post-test probabilities. A positive IL-6 test result increased the post-test probability of LONS to 67%, whereas a negative result decreased it to 6%. These results suggest that IL-6 can meaningfully alter clinical decision-making in NICU settings, supporting its utility in both ruling in and ruling out sepsis.

While these results support the diagnostic utility of IL-6, claims of “reliability” should still be moderated due to the observed variability in test characteristics across studies, despite the low statistical heterogeneity (*I*^2^ = 13.8%). This variability may be attributed to several methodological and clinical factors. First, the wide range of IL-6 cut-off values likely influenced diagnostic performance. While lower thresholds typically increase sensitivity at the expense of specificity, and higher thresholds do the opposite, this expected trend was not consistent across the included studies. For instance, some studies using low cut-offs (e.g., 7 pg/mL) achieved unexpectedly high specificity, whereas others using high cut-offs reported excellent sensitivity [19, 49]. Second, differences in patient populations—including gestational age, birth weight, and clinical status—may alter baseline IL-6 levels and affect performance. Third, the timing of IL-6 measurement relative to symptom onset could impact its diagnostic accuracy. Lastly, although blood culture was used as the reference standard across studies, variations in culture techniques, blood volume drawn, and prior antibiotic exposure may have influenced the confirmation of sepsis, thereby affecting sensitivity and specificity estimates.

Our meta-analysis aligns with the previous findings of Eichberger et al., who reported pooled sensitivity and specificity of 88% and 78%, respectively, from 15 studies [52]. Their subgroup analysis showed higher sensitivity in preterm (87%) versus term/mixed infants (82%), while specificity remained the same (86%). Unlike Eichberger et al., our meta-analysis includes more recent studies and focuses on IL-6 accuracy alone, excluding combined biomarkers. Sun et al. conducted a meta-analysis on early-onset sepsis, reporting pooled sensitivity and specificity of 88% and 82% [53]. Another meta-analysis focused on sepsis in neonates with premature rupture of membranes found an overall sensitivity of 85% and specificity of 88%, with early/late-onset cases showing higher sensitivity (93% vs. 80%) and specificity (98% vs. 86%) than early-onset cases [54].

The diagnostic performance of IL-6 appears to be better than that of conventional inflammatory markers, including CRP and PCT, with emphasis mainly on its earlier rise. Pons et al. reported the accuracy of various biomarkers in LONS: PCT, IL-6, IL-10, IL-27, cluster of differentiation 14 (CD14), neutrophil gelatinase-associated lipocalin (NGAL), interferon gamma-induced protein 10 (IP-10), pentraxin 3 (PTX3), and lipopolysaccharide-binding protein (LBP), gelsolin, and calprotectin. They highlighted IL-6, IL-10, and NGAL as the best biomarkers, with area under the curve of 86%, 84.5%, and 82.9%, respectively [20]. A recent study by Chen et al. compared the diagnostic power of IL-6, IL-8, serum amyloid A (SAA), CRP, and PCT [33]. They reported that IL-6, IL-8, and SAA, unlike PCT or CRP, had better diagnostic accuracy. PCT combined with IL-6 gave better accuracy for term infants with sepsis [33]. Similarly, Cui et al. reported sensitivity and specificity higher than 85% when PCT or CRP was combined with IL-6 [34]. Degirmencioglu et al. observed the highest accuracy parameters of IL-6 (AUC of 0.959, 94.4% sensitivity, and 78.2% specificity) compared to presepsin (AUC 0.939, 88.9% sensitivity and specificity), CRP (AUC 0.850, 72.2% sensitivity, 81.5% specificity), and Fetuin-A (AUC 0.612, 72.2% sensitivity, 48.1% specificity) [35]. On the contrary, a recent study reported higher accuracy of CRP and PCT over IL-6 when using point-of-care testing [18]. This may be attributed to the rapid turnaround and reduced sample handling in POC testing, which minimizes pre-analytical variability and allows for more timely and potentially more accurate detection.

Defining the optimal cutoff value of IL-6 is continuously investigated. Kung et al. reported that the use of IL-6 cutoff of 80 picograms per milliliter (pg/mL) on day 1, 40 pg/mL on days 2 to 7, and 30 pg/mL after day 7 produced sensitivity and specificity of 75% and 81% for culture-confirmed sepsis, respectively [40]. As previously discussed, the IL-6 cutoff values did not follow a consistent trend across studies. Chen et al. employed a cutoff level of 44.487 pg/mL for premature infants and 43.422 pg/mL for term infants, reporting sensitivities of 68% and 60%, while specificities were 95% and 96%, respectively [33]. Rupin et al. set a far higher cutoff of 200 pg/mL, achieving 100% sensitivity against a specificity of 78% [19]. The highest accuracy metrics were reported by Santos et al., Sladir et al., and Tunk et al., using cutoff values of 7 and 10 pg/mL, with sensitivity and specificity over 90% [36, 47, 49]. This highlights the superiority of lower cutoff values in diagnosing LONS. However, Goyal et al. chose a cutoff of 7 pg/mL, reporting a sensitivity of 100% but a considerably lower specificity of 34.48% [18].

Although the included studies in the current meta-analysis varied in their IL-6 cut-off values, we found no evidence of a significant threshold effect based on the Spearman correlation between the logit of sensitivity and the logit of 1-specificity. This suggests that the variation in diagnostic accuracy is unlikely to be solely due to threshold differences. While we considered incorporating the IL-6 cut-off value as a covariate in an HSROC model, the analysis was not pursued due to the highly skewed distribution of thresholds—with over two-thirds of studies using values ≥ 20 pg/mL—which would have compromised the interpretability and power of such a model. Therefore, while threshold variability remains a plausible contributor to heterogeneity, it could not be formally explored through stratified analysis in this meta-analysis.

Our work has several strengths, including the use of robust analytic methods such as bivariate random-effects modeling, formal assessment for threshold effect, and pre-specified subgroup analyses. In addition, the use of Fagan analysis is a strength of this review, as it bridges statistical accuracy with bedside utility—especially relevant in high-stakes neonatal care. The change in post-test probabilities demonstrates that IL-6 can enhance the clinician’s confidence in decision-making, particularly when used alongside clinical judgment and other markers. Lastly, we applied the GRADE approach for diagnostic test accuracy to evaluate the certainty of evidence for both sensitivity and specificity—an essential yet often overlooked step in diagnostic meta-analyses. This enhances the interpretability and credibility of our findings.

Several limitations must be acknowledged. First, although IL-6 cutoff values varied widely across the included studies, we found no significant threshold effect based on Spearman correlation, and the low statistical heterogeneity (*I*^2^ = 13.8%) suggests that this variability did not meaningfully affect the pooled estimates. However, due to the skewed distribution of thresholds, we were unable to perform a formal subgroup analysis using HSROC modeling. Second, the possibility of publication bias cannot be excluded, as suggested by the asymmetry in the trim-and-fill funnel plot, which may lead to overestimation of diagnostic accuracy. Third, most included studies were conducted in middle- and high-income countries, limiting generalizability to lower-resource settings where neonatal sepsis epidemiology and care infrastructure may differ. Also, the focus on English-language studies represents a potential limitation, as it may have excluded relevant research published in other languages. Despite these limitations, the diagnostic performance of IL-6 remained consistent and robust across multiple analyses. Future research should focus on validating standardized cutoff values and evaluating IL-6 performance in broader clinical and geographical contexts.

## Conclusion

IL-6 is a biomarker with high sensitivity and specificity for late-onset neonatal sepsis, with good rule-in and rule-out potential. Its rapid rise during infection makes it clinically useful and practical for detecting early sepsis. Further research should aim to identify and define an optimal threshold and validate IL-6 performance in various neonatal populations to improve reliability in clinical practice.

## Supplementary Information

Below is the link to the electronic supplementary material.

Supplementary Material 1 (DOCX 90.9 KB)

## Data Availability

The datasets generated during and/or analyzed during the current study are available from the corresponding author on reasonable request.
