# Supplementary material for: Diagnostic accuracy of interleukin-6 (IL-6) as a significant biomarker in late-onset neonatal sepsis: an updated systematic review and meta-analysis
Source: Eur J Pediatr. 2025 Sep 2;184(9):587. doi: 10.1007/s00431-025-06409-w (PMC12405383; doi:10.1007/s00431-025-06409-w)

**Table S1:** Search strategy for each database.

| **Database** | **Search Strategy** |
| --- | --- |
| **PubMed** | (Interleukin-6[All Fields] OR IL-6[All Fields]) AND (Sepsis[All Fields] OR Septic*[All Fields] OR "Blood infection*"[All Fields] OR "Blood poisoning"[All Fields] OR SIRS[All Fields]) AND (Newborn*[All Fields] OR Neonat*[All Fields] OR Infant*[All Fields] OR Natal[All Fields]) |
| **Scopus** | ( TITLE-ABS-KEY ( ( Interleukin-6 OR IL-6 ) ) AND TITLE-ABS-KEY ( ( Sepsis OR Septic* OR "Blood infection*" OR "Blood poisoning" OR SIRS ) ) AND TITLE-ABS-KEY ( ( newborn* OR neonat* OR infant* OR natal ) ) ) |
| **Web of Science (WoS)** | TS=(Interleukin-6 OR IL-6) AND TS=(Sepsis OR Septic* OR "Blood infection*” OR "Blood poisoning" OR SIRS) AND TS=(newborn* OR neonat* OR infant* OR natal) |
| **Cochrane** | (Interleukin-6 OR IL-6) in Title Abstract Keyword AND (Sepsis OR Septic* OR Blood infection* OR Blood poisoning OR SIRS) in Title Abstract Keyword AND (newborn* OR neonat* OR infant* OR natal) in Title Abstract Keyword |
| **Embase** | ('interleukin 6':ti,ab,kw OR 'il 6':ti,ab,kw) AND (sepsis:ti,ab,kw OR septic*:ti,ab,kw OR 'blood infection*':ti,ab,kw OR 'blood poisoning':ti,ab,kw OR sirs:ti,ab,kw) AND (newborn*:ti,ab,kw OR neonat*:ti,ab,kw OR infant*:ti,ab,kw OR natal:ti,ab,kw) |

**Table S2:** Quality assessment of the included studies using QUADAS-2 tool.

| **ID** | **Patient Selection** | **Index Test(s)** | **Reference Standard** | **Flow and Timing** | **Overall Risk** |
| --- | --- | --- | --- | --- | --- |
| Chen 2025 | low | low | low | low | low |
| Santos 2024 | low | low | low | low | low |
| Goyal 2024 | low | low | low | low | low |
| Rupin 2024 | low | low | high | high | high |
| Gatseva 2023 | low | moderate | high | low | moderate |
| Kung 2023 | high | low | low | high | high |
| Pons 2023 | low | low | high | low | moderate |
| Cui 2021 | low | low | low | low | low |
| Değirmencioğlu 2019 | low | low | low | low | low |
| Saldir 2015 | low | low | high | low | moderate |
| Tunc 2014 | low | low | high | low | moderate |
| Lusyati 2013 | low | low | low | low | low |
| Maaboud 2012 | low | low | high | low | moderate |
| Raynor 2012 | moderate | low | moderate | low | moderate |
| Hotoura 2011 | low | low | low | low | low |
| Sarafidis 2010 | low | low | moderate | low | moderate |
| Ng 2007 | low | low | low | low | low |
| Maciolek 2006 | low | low | low | low | low |
| Arnon 2005 | low | low | low | low | low |
| Gonzalez 2003 | low | low | low | low | low |
| Ng 2002 | low | low | low | low | low |
| Ng 1997 | low | low | low | low | low |

**Table S3:** GRADE assessment of certainty of bivariate model results.

| **Outcome** | **No of studies (No of patients)** | **Study design** | **Factors that may decrease the certainty of evidence** | | | | | **Effects per 100 patients tested** | **Test accuracy CoE** |
| --- | --- | --- | --- | --- | --- | --- | --- | --- | --- |
|  |  |  | **Risk of bias** | **Indirectness** | **Inconsistency** | **Imprecision** | **Publication bias** | **pre-test probability of 41.2%** |  |
| **True positives** (patients with Late-Onset Sepsis) | 20 studies 772 patients | cohort & case-control type studies | not serious | not serious | not serious | not serious | publication bias is strongly suspected^a^ | 35 (33 to 37) | ⨁⨁⨁◯ Moderate^a^ |
| **False negatives** (patients incorrectly classified as not having Late-Onset Sepsis) |  |  |  |  |  |  |  | 6 (4 to 8) |  |
| **True negatives** (patients without Late-Onset Sepsis) | 20 studies 1101 patients | cohort & case-control type studies | not serious | not serious | not serious | not serious | publication bias is strongly suspected^a^ | 50 (46 to 53) | ⨁⨁⨁◯ Moderate^a^ |
| **False positives** (patients incorrectly classified as having Late-Onset Sepsis) |  |  |  |  |  |  |  | 9 (6 to 13) |  |

a. Trim and fill funnel plot for diagnostic odds ratio of included studies showed an asymmetrical pattern with estimated publication bias for several studies with comparatively small effect sizes.

**Table S4:** Table 1: Reported diagnostic performance of IL-6 as per included studies (n: 20 studies)

| **ID** | **Sensitivity (95% CI)** | **Specificity (95% CI)** | **DOR (95% CI)** |  |
| --- | --- | --- | --- | --- |
| Chen 2025 | 64 (55-72) | 96 (91-98) | 41.19 (15.61-108.65) |  |
| Santos 2024 | 96 (87-99) | 91 (72-97) | 255 (33.59-1935.84) |  |
| Goyal 2024 | 99 (94-100) | 34 (25-45) | 86.28 (5.16-1443.06) |  |
| Gatseva 2023 | 76 (55-89) | 71 (55-84) | 8 (2.31-27.74) |  |
| Kung 2023 | 75 (66-82) | 81 (79-83) | 12.97 (8.25-20.39) |  |
| Pons 2023 | 90 (79-96) | 79 (72-85) | 34.79 (12.77-94.73) |  |
| Cui 2021 | 88 (79-93) | 90 (75-97) | 66.18 (17.2-254.69) |  |
| Değirmencioğlu 2019 | 93 (77-98) | 83 (67-92) | 62.83 (11.65-339.02) |  |
| Saldir 2015 | 93 (79-98) | 95 (76-99) | 266 (22.5-3145.19) |  |
| Tunc 2014 | 97 (83-99) | 95 (76-99) | 551 (32.47-9351.25) |  |
| Lusyati 2013 | 93 (69-99) | 90 (60-98) | 117 (6.44-2124.63) |  |
| Maaboud 2012 | 90 (78-95) | 92 (80-97) | 106.07 (23.73-474.08) |  |
| Hotoura 2011 | 97 (78-100) | 74 (59-85) | 101.67 (5.61-1842.69) |  |
| Sarafidis 2010 | 81 (64-91) | 81 (60-92) | 17.71 (4.34-72.33) |  |
| Ng 2007 | 82 (68-90) | 82 (74-88) | 20.48 (8.27-50.67) |  |
| Maciolek 2006 | 68 (51-80) | 77 (58-89) | 6.94 (2.21-21.78) |  |
| Arnon 2005 | 79 (64-89) | 88 (80-94) | 28.75 (10.12-81.7) |  |
| Gonzalez 2003 | 75 (41-93) | 68 (46-85) | 6.5 (1-42.17) |  |
| Ng 2002 | 78 (63-89) | 92 (85-96) | 42.98 (14.32-128.99) |  |
| Ng 1997 | 89 (77-95) | 96 (88-99) | 216 (39.86-1170.64) |  |
| Correlation between sensitivity and FPR = rho 0.074 (p-value: 0.757) | | | | |

**Table S5:** Reitsma’s bivariate model for pooling IL-6 diagnostic performance.

| **Term** | **Estimates from Reitsma bivariate model** | **Sensitivity analysis excluding high ROB studies (included n: 19 studies)** | **Sensitivity analysis excluding non-prospective cohort studies (included n: 15 studies)** |
| --- | --- | --- | --- |
|  | **Estimate (95% CI)** | | |
| Sensitivity | 0.852 (0.8-0.893) | 0.861 (0.807-0.902) | 0.861 (0.79-0.911) |
| False pos. rate | 0.159 (0.11-0.225) | 0.157 (0.106-0.226) | 0.158 (0.099-0.243) |
| Specificity | 0.841 (0.775-0.89) | 0.843 (0.774-0.894) | 0.842 (0.757-0.901) |
| Positive LR | 5.44 (3.81-7.64) | 5.61 (3.85-8.03) | 5.59 (3.61-8.47) |
| Negative LR | 0.18 (0.13-0.24) | 0.17 (0.12-0.23) | 0.17 (0.11-0.24) |
| Diagnostic odds ratio | 31.4 (18.6-49.8) | 34.6 (20.1-55.5) | 34.5 (18.6-58.7) |
| AUC | 91% | 91.5% | 91.6% |
| I^2 | 13.8% | 13.9% | 19.2% |

**Figure S1:** Fagan nomogram.


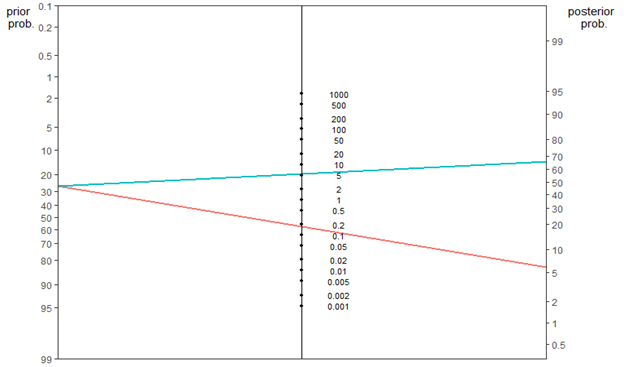


Figure S2: Trim and fill-based funnel plot for assessment of publication bias.


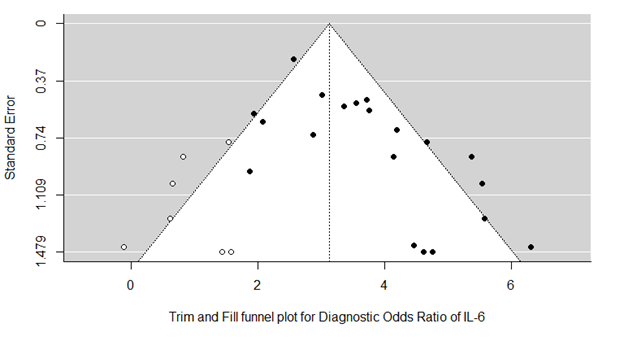

Supplement: Supplementary file 1 — Supplementary Material 1 (DOCX 90.9 KB) [file 431_2025_6409_MOESM1_ESM.docx]
